# Complete Bilateral Ossification of the Arytenoid Cartilages With Minimal Involvement of the Laryngeal Skeleton: A Unique Anatomical Variation

**DOI:** 10.7759/cureus.78525

**Published:** 2025-02-04

**Authors:** Nymfodora Malkidou, Aliki Fiska, Katerina Vassiou

**Affiliations:** 1 Anatomy, Anatomy Laboratory, Medical School University of Thessaly, Larissa, GRC; 2 Anatomy, School of Medicine-Democritus University of Thrace, Alexandroupolis, GRC

**Keywords:** anatomical variation of arytenoid cartilage, complete arytenoid cartilage ossification, laryngeal cartilage ossification, larynx ossification pattern, non-ossified cricoid cartilage

## Abstract

The arytenoid cartilages are distinguished by their mobility and unique mixed composition of hyaline and elastic cartilage. Though laryngeal cartilage ossification is not rare, and its pattern is well-studied, sole complete and bilateral arytenoid cartilage ossification is an aberration. An 84-year-old woman, with no history of voice or breathing issues, was brought to the emergency department for a head-and-neck trauma following a fall. CT scans revealed a subdural hematoma and, incidentally, a bilateral ossification of the arytenoid cartilages, sparing the vocal processes but involving complete ossification of the hyaline part and the tip. The cricoid cartilage was non-ossified, and the thyroid depicted minor ossification. We present a unique, previously undocumented case of both arytenoid cartilages simultaneously ossified in a patient without laryngeal pathology. In previous studies on laryngeal cartilage ossification during aging, no cases have demonstrated either bilateral complete ossification of the arytenoid cartilages or arytenoid ossification occurring before that of the thyroid and cricoid cartilages. Ossification is an age-related process; our case’s ossification pattern does not align with the expected final stage. The involvement of the arytenoid tip is also an exceptional observation. The unique anatomical variation in the laryngeal ossification pattern underlines that arytenoid cartilage ossification may be complete and bilateral and precede that of the thyroid or cricoid cartilage. It could be the only notable feature on a neck scan of a patient with no history of laryngeal disorders.

## Introduction

The arytenoid cartilages are pyramidal, three-sided structures situated on the posterior upper margin of the cricoid cartilage. They stand out from other laryngeal cartilages because of their high mobility and their composition of both hyaline and elastic cartilage. The thyroid and cricoid consist only of hyaline cartilage, which typically starts to ossify after the age of 18, whereas epiglottis, made entirely of elastic cartilage, does not undergo ossification [1-2].

The calcification of the laryngeal cartilages is not rare, and it has been thoroughly investigated. Hartley et al conducted the first comprehensive study of laryngeal cartilage ossification patterns, analyzing the largest case series of their era. They established that thyroid cartilage ossification is usually the most prominent and the first to appear, starting at the inferior horns, with the superior horns typically ossifying at a later stage [3]. Cricoid cartilage ossification may occur concurrently or follow the thyroid changes, and the arytenoids usually exhibit none or minor ossification, occurring later. The foci appear on their apices, bodies, and muscular processes, but the vocal processes and the tips are consistently unaffected.

We present the case of an 84-year-old woman without any laryngeal pathology, who was incidentally found to exhibit bilateral ossification of the entire hyaline part of the arytenoid cartilages, combined with minimal ossification of the thyroid cartilage and no ossification of the cricoid. To the best of our knowledge, this is the first documented case of this exceptional event. This anatomical variation was described using the Anatomical Quality Assurance (AQUA) checklist (Appendices) [4].

## Case presentation

An 84-year-old woman was brought to the emergency department of a private hospital in Larissa following an accidental fall at home resulting in head and neck trauma. She was on anticoagulant therapy with apixaban for stroke prevention due to atrial fibrillation. Her family reported an episode of acute loss of consciousness after the fall. Given her medical history and symptoms, an urgent computed tomography scan of the brain and cervical spine was performed.

**Figure 1:**
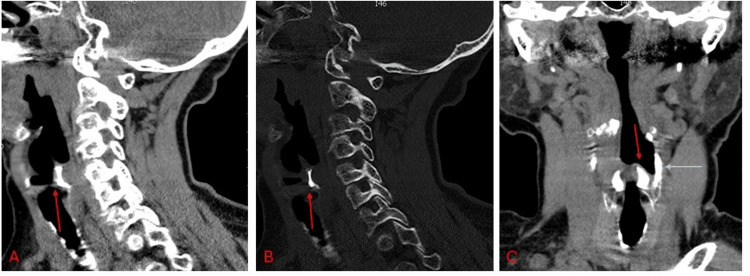
Arytenoid cartilage ossification with minimal involvement of the laryngeal skeleton Constructed CT of the neck, sagittal plane, shows complete ossification of the arytenoid cartilage, excluding the vocal process. A. with soft tissue density (red arrow) and B. with bone density (red arrow). C. Constructed CT of the neck, coronal plane, with soft tissue density, shows complete ossification of the arytenoid cartilages (red arrow), ossification of the superior thyroid horns (grey arrow), and no sign of cricoid involvement, highlighting the ossification at the tip of the arytenoids.

Brain CT revealed a subdural hematoma without midline shift, likely accounting for her loss of consciousness without requiring intubation. Cervical spine CT indicated cervical vertebral osteopenia. Incidentally, the neck visceral space showed bilateral symmetric ossification of the arytenoid cartilages sparing the vocal processes. Limited high-density calcifications were noted in the superior horns of the thyroid cartilage, the thyroid angle, and the thyrohyoid ligament. The remainder of the thyroid cartilage and the entire cricoid cartilage exhibited minimal or no ossification. Notably, the ossified arytenoid cartilage demonstrated on CT the same high density as the mandible (Figure 1).

Upon our inquiry, her family reported she had no history of voice changes, breathing difficulties, or other significant otolaryngological issues. However, due to her impaired level of consciousness, a comprehensive ENT evaluation, including a detailed assessment of laryngeal function and voice, could not be conducted at that time. Following the resolution of the hematoma, the patient resumed her daily activities. As she did not experience any voice or breathing problems, an ENT examination was not considered necessary. The subdural hematoma resolved spontaneously after 14 days, and the patient was discharged after 5 days without any changes to her medication. Regarding the finding on the CT scan of the larynx, an appointment was scheduled with an otolaryngologist, but the patient did not attend. While the finding may be incidental and potentially represent an anatomical variation, the absence of an ENT examination leaves the possibility of an underlying laryngeal pathology, which has not yet manifested, to be carefully considered.

## Discussion

Bilaterally ossified arytenoid cartilages, involving complete ossification of the hyaline part, are remarkable, especially with no ossification observed in the cricoid and minimal osseous changes in the thyroid cartilage (Figure 1).

The abnormal ossification pattern of the laryngeal skeleton of our case is unique according to previous studies. Zan et al. reviewed 972 computed tomography (CT) scans to evaluate arytenoid ossification and found no evidence of simultaneous bilateral ossification of the arytenoid cartilages [5]. Yeager et al., who investigated 30 cases of laryngeal cartilage ossification using CT scans, found no evidence of complete arytenoid involvement [6]. Hartley et al., examining 516 neck X-rays, found no signs of ossification in the arytenoids when the thyroid and cricoid cartilages remained unossified [3].

Kahane noted that the ossification process is typically completed by the eighth decade, even if the ossification is partial [7]. Turk et al. in their radiographic study confirmed that the ossification of laryngeal cartilages is age-related; they established specific age-based stages for each cartilage [8]. Given our patient’s old age, we may assume that the laryngeal ossification process is ending, and while the ossification pattern of the arytenoids aligns with the expected final phase, that of the thyroid and cricoid cartilages does not.

However, this finding could either represent an anatomical variation or potentially mask a latent laryngeal pathology that has yet to manifest. Recognizing such cases is crucial, as demonstrated by Morreels et al., where a unilateral ossified arytenoid cartilage was misidentified as a foreign body, leading to the patient undergoing unnecessary surgery [9].

As a concluding remark, from a histologic and physiological perspective, the arytenoid elastic cartilages are located at two sites: the vocal process to facilitate movement and the tip to act as a cushion, protecting the mucosa from mechanical damage [2, 10]. These are typically non-ossifying areas. Interestingly, in our case, while ossification was absent in the vocal process of the arytenoid cartilages, it was observed at the tip of both of them (Figure 1C). This raises the question of whether the vocal process is the only component entirely composed of elastic cartilage.

## Conclusions

Our case highlights the complete, bilateral ossification of the arytenoid cartilages, combined with minimal thyroid and the absence of cricoid cartilage ossification. This unique variation of the laryngeal ossification pattern was accompanied by ossification at the tip of the arytenoid cartilages, an area typically spared due to comprising of elastic cartilage. Our findings suggest that arytenoid cartilage ossification may precede that of the thyroid or cricoid cartilage and could be the only notable feature on a CT scan of the neck’s visceral space in a patient without laryngeal pathology. This case could serve as a reference and a basis for future research into laryngeal degeneration processes.

## Appendices

**Table 1:** Anatomical Quality Assurance (AQUA) Checklist Tomaszewski et al. [4]

Checklist Component	#	Description and Recommendation	Present/Section in Current Article
Title			
Title	1	Identify the main objective or key characteristic of the study in the title.	Yes/Title
Abstract			
Structured Summary	2	Provide a clear and structured summary of the study with emphasis on the aims, methodology, key findings, and conclusions directly supported by study findings.	Yes/Abstract
Introduction			
Background / Rationale	3	Provide a rationale for the study including a concise, updated scientific background, appropriately referenced. Identify any relevant knowledge gaps to support the study rationale.	Yes/Introduction
Objective	4	Indicate clearly the main objective(s) of the study, and state any hypotheses to be tested.	Yes/Introduction
Methodology			
Study Design and Fundamentals	5	Provide precise details with respect to the design and fundamentals of the study, including but not limited to the following: Study design: prospective, retrospective, cross-sectional, etc. Study type: cadaveric (e.g. formalin fixed or fresh frozen), imaging, intraoperative, etc.	Yes/Case Presentation
Setting	6	Describe clearly the location where the study was conducted and dates (month/year) between which the data were collected.	Yes/Case Presentation
Sample Size	7	When appropriate, statistical power analysis should be used to calculate sample size or effect size. If relevant, justification for the study sample size should be briefly stated.	Not Applicable
Subjects	8	Define clearly the eligibility criteria and methods of subject selection and inclusion, with details of the baseline and demographic selection criteria of the subjects (age, sex, healthy or diseased etc.) included in the study.	Yes/Case Presentation
Reference Standard	9	Define clearly and accurately all anatomical definitions (normal anatomy, variations, classifications, etc.) by which data will be collected, analyzed, and compared. Citations should be included when appropriate.	Yes/Case Presentation
Outcomes and/or Parameters	10	Define clearly the outcomes and parameters (e.g. prevalence of a variation, mean length and diameter of a structure, etc.) assessed in the study. When present, confounders should be clearly stated.	Yes/Case Presentation
Measurement and Assessment	11	Indicate clearly the group of subjects included in each measurement/assessment (source of data). Provide clear details about the methods of measurement/assessment of each outcome and/or parameter (e.g. reference points for length measurements, internal or external diameter, etc.).	Not Applicable
Modality	12	Describe clearly the materials, equipment, and instruments used (with manufacturer/supplier details) to conduct the specific study design.	Yes/Case Presentation
Technique	13	Describe precisely the methods (e.g. dissection technique, image reconstruction, etc.) applied in the study to allow for reproducibility. Relevant details (profession, years of experience) regarding the individual(s) performing the technical aspect of the study are recommended.	Yes/Case Presentation
Bias	14	Identify any potential source of bias and, when present, describe measures implemented to assess the risk of bias.	Not Applicable
Statistical Approach	15	Describe all statistical methods for analyzing the data, including those of confounders. Statistical methods for additional analyses (e.g. subgroup/sensitivity analyses), when performed, should be described.	Not Applicable
Ethics	16	Provide the details of compliance with ethical guidelines, including the name of the review board or agency, approval number, and date. AQUA endorses the Helsinki Declaration and its later amendments. When appropriate, details of written, informed consent should be clearly stated.	Yes/Case Presentation
Results			
Subjects	17	Report the number of subjects included in the study, including data on their baseline and demographic characteristics. When needed, provide reason(s) and data on characteristics of the subjects excluded from the study at any stage.	Yes/Case Presentation
Main Results	18	Provide unaltered/non-manipulated summary data (number [percentage]) or estimates (with confidence intervals and values of consistency when applicable) from the analyses performed. Tabular presentation of the results is highly recommended.	Yes/Case Presentation
Descriptive Anatomy	19	Present clear and comprehensible figures (i.e. images, illustrations, diagrams, etc.), labeled as appropriate, to explain the results where needed AND describe clearly any anatomical findings that could be ambiguous, questionable, or atypical. New classifications of anatomical variations should be complemented by representative figures and corresponding dissection/imaging photographs.	Yes/Case Presentation
Confounders	20	Present precise data from assessment/measurement of confounders, if any.	Not Applicable
Additional analyses	21	Provide clear results of additional analyses (e.g. subgroup/sensitivity analyses), if performed. Tabular presentation of the results is highly recommended.	Not Applicable
Discussion			
Key Findings	22	Include summary of key evidence/findings from the study pertaining to the rationale/objectives of the study. No new study results should be presented in the discussion.	Yes/Discussion
Interpretation and Comparison(s)	23	Provide comprehensive (but judicious) interpretation of the results from the study, and comparison and/or reference to the results from other studies on the topic, appropriately cited. Meaningful clinical impact/significance of the findings from the study should be discussed where relevant.	Yes/Discussion
Implication(s)	24	State briefly the implications of the findings or potential areas of the study that require further research.	Yes/Discussion
Limitation(s)	25	Discuss briefly limitations of the study at any stage, including risk of bias, potential confounders, or intraobserver and/or interobserver variability.	Yes/Discussion
Conclusions			
Summary	26	Summarize the key information (i.e. “take-home message”) directly supported by the findings/evidence from the study.	Yes/Conclusions
Other Information			
Acknowledgement	27	Acknowledge individual(s), institution(s), or third parties who significantly contributed to the study.	Not Applicable
Conflict of interest	28	Disclose any conflicts of interests related to the study for all contributing authors.	Yes/Disclosures
Funding	29	Describe sources of funding for the study and any other support.	Yes/Disclosures
